# Feasibility study to inform the design of a randomised controlled trial to eradicate *Pseudomonas aeruginosa *infection in individuals with Cystic Fibrosis

**DOI:** 10.1186/1745-6215-11-11

**Published:** 2010-02-05

**Authors:** Helen R Hickey, Ashley P Jones, Warren Lenney, Paula R Williamson, Rosalind L Smyth

**Affiliations:** 1 Medicines for Children Research Network Clinical Trials Unit, University of Liverpool, UK; 2 University Hospital of North Staffordshire, Staffordshire, UK; 3 Division of Child Health, School of Reproductive and Developmental Medicine, University of Liverpool, Alder Hey Children's Foundation Trust, Liverpool, L12 2AP, UK

## Abstract

**Background:**

There are controversies about the most effective treatment to eradicate first growth of *Pseudomonas aeruginosa (P aeruginosa) *from the lower airways of patients with cystic fibrosis (CF). UK guidelines recommend oral treatment, but some advocate intravenous (IV) treatment. The objective of this study was to assess the feasibility of conducting a randomised controlled trial comparing two treatment strategies to eradicate *P aeruginosa *in CF patients.

**Methods/Principal Findings:**

Two surveys were conducted. Survey [1] included clinicians who were responsible for the treatment of individuals with CF, to assess their clinical practice, opinions and numbers of potentially eligible patients. Survey [2] included adults and young people aged 13 years or more with CF and parents of children with CF aged less than 13 years, identified at six UK CF centres, who fulfilled eligibility criteria for the proposed clinical trial, to assess their views about the interventions and their willingness to participate in the trial. Generally clinicians treat first or new growth of *P aeruginosa *with oral antibiotics, but 90% reported that they would consider IV treatment of first isolation of *P aeruginosa*. 74% of clinicians would consider recruiting their patients and 45% of consumers would consider entry for themselves or their children into a trial comparing oral with intravenous antibiotics. The median rate per annum for first or new growths of *P aeruginosa *in adults was 3% (range 1% to 9%) and in children was 10% (range 3% to 23%). If the trial was conducted across the UK, with a consent rate of 45%, then the number of eligible patients per annum who would be willing to take part in a study would be approximately 41 adults and 203 children.

**Conclusions:**

This work demonstrates the importance of feasibility studies in preparation for multicentre clinical trials. It confirmed the uncertainty amongst clinicians and patients about the clinical question, enabled assessment of the number of potentially eligible patients, the proportion of patients and clinicians prepared to participate and aspects of trial design which might encourage this. It showed that a clinical trial was feasible, but only if patients were recruited from across United Kingdom.

## Introduction

Cystic fibrosis (CF) is the most common life-limiting inherited disease in Caucasian populations, where the prevalence is around one in 2000 live births. The primary cause of death in people with CF is respiratory failure resulting from chronic pulmonary infection[1]. Acquisition of chronic infection of the airways with *Pseudomonas aeruginosa (P aeruginosa) *has been consistently shown to be associated with shortened survival[2,3]. Because of this, in most CF centres, identification of *P aeruginosa *from the lower airways of a CF patient, previously considered free of this infection, is treated with therapy designed to eradicate the organism[4]. However evidence for the overall effectiveness of this strategy, or the optimal treatment regimen to achieve this outcome is lacking[5].

In the UK, when a patient has a first respiratory culture in which *P aeruginosa *is isolated, the UK Cystic Fibrosis Trust (UK CF Trust) recommends commencement of eradication therapy in the form of nebulised colistin and oral ciprofloxacin for three weeks, following which cultures should be repeated; if the repeat culture is negative no further colistin or ciprofloxacin is given[6]. However, some clinics continue colistin and ciprofloxacin for three months, in line with practice in Denmark[4]. Whilst acknowledging the lack of evidence, the guidelines present a number of alternatives for management, should this initial treatment be unsuccessful in eradicating *P aeruginosa; *these include intravenous therapy, for two weeks, with two anti-pseudomonal antibiotics.

The most reliable method of addressing the lack of evidence would be to conduct a randomised controlled trial (RCT), using the current standard protocol of oral ciprofloxacin plus nebulised colistin compared with intravenous anti-pseudomonal antibiotics, both groups receiving, in addition, nebulised colistin for three months. IV therapy is invasive, thus there are concerns about the acceptability of this trial to patients with CF and, for children, their parents. Clinicians may also not be in equipoise about this treatment and because CF is a relatively rare disease, it may be impossible to recruit adequate numbers of patients with CF, who fulfil the inclusion criteria, to a clinical trial. A major UK funding body, the NIHR Health Technology Assessment Programme, were considering commissioning the proposed trial and commissioned this feasibility study to:

• Conduct a survey of current practice for treatment of *P aeruginosa *infection.

• Conduct a survey of attitudes of clinicians, parents (and patients if old enough) towards the proposed RCT.

• Estimate the number of cystic fibrosis patients who are currently not colonised with *P aeruginosa *and the number likely to become positive each year (and therefore eligible for the proposed RCT).

## Methods

A consortium of Directors from five UK CF centres and staff from the Medicines for Children Research Network Clinical Trials Unit (MCRN CTU) collaborated to design survey questionnaires relevant to two target groups; CF clinicians and consumers who were potential participants for the proposed RCT, or parents of potential participants. Prior to commencing, confirmation was received from the UK Central Office for Research Ethics Committees (now National Research Ethics Service) that ethical approval was not required.

In September 2006, all UK CF centre clinicians (63) who were registered with the UK CF Trust were contacted and invited to complete a questionnaire (Additional file 1). Individuals were initially approached by e-mail in the first instance and were invited to complete an electronic survey, within three weeks, after which time electronic access was disabled. Those who failed to complete and submit an electronic questionnaire in three weeks were sent a paper copy and asked to complete it. All respondents were anonymous to the study team, however a unique code assigned by the UK CF Trust to questionnaires completed online enabled the population type (adult or paediatric) to be reported (this information was not available for those questionnaires that were received by post). The questionnaire provided details about the proposed clinical trial, including the eligibility criteria for patients, the interventions in the two treatment regimens and two suggested primary outcome measures: time to next growth of *P aeruginosa *following successful eradication and eradication of *P aeruginosa *from any lower respiratory tract culture over a two year period. The questionnaire asked about clinicians' current management of first or new isolation of *P aeruginosa*, their views about the certainty of evidence for these strategies and whether they would be prepared to recruit patients to the proposed trial. Finally, to provide an estimate of the number of potentially eligible patients presenting per annum in the UK, clinicians were asked about the population size of their CF clinic, the number of patients who have never had a lower respiratory tract culture or who are not colonised with *P aeruginosa *and how many patients present with first or new growths of *P aeruginosa *per annum.

In parallel with the clinician survey, the consumer questionnaire was developed. This delivered a description of the proposed trial in a similar format to that which would be used in a patient information leaflet to inform consent. A draft of the consumer questionnaire was opportunistically discussed in clinic with children, parents and the multidisciplinary CF team at Alder Hey Children's Foundation Trust, Liverpool and revised to reflect comments.

Patients and parents of children who would fulfil the eligibility criteria for the proposed trial were invited to complete the consumer survey (Additional file 2). These were either patients with CF who were ≥ 13 years of age or the parent of a child with CF aged < 13 years. In addition, they must either (a) never have isolated *P aeruginosa *from a respiratory culture or (b) have previously isolated *P aeruginosa*, undergone eradication therapy and have had at least three consecutive negative respiratory cultures over a six month period to indicate eradication therapy had been successful. The target recruitment for the consumer survey was at least 50 parents and 50 young people or adults with CF. Consumer questionnaires were delivered to six participating UK CF centre sites, for distribution in routine outpatient clinics between 24 July and 30 October 2006. These centres were purposively sampled, based upon the judgment of the lead investigators, as being representative of UK adult and paediatric CF clinics that would ultimately be recruitment centres for inclusion in the definitive trial. These centres were able to provide target recruitment of adults and children with CF in this time period, with clinics typically seeing 15 to 20 patients per week. Adults and young people aged 13 years or more with CF and parents of children with CF aged less than 13 years who would fulfil eligibility criteria for the proposed clinical trial were approached consecutively as they attended the appropriate clinic until target study accrual was reached. Questionnaires were generally distributed and collected by clinical nurse specialists during these out-patient clinics, with participants encouraged to complete and return the survey before leaving the hospital. Participants were asked to complete the questionnaire once only, and only one questionnaire was completed per family unit.

Respondents were provided with envelopes within which to seal completed surveys as an assurance that their answers remained confidential. The nurse specialists were responsible for returning the sealed envelopes to the MCRN CTU, maintaining anonymity of respondents. The questionnaires explored consumer attitudes to the envisaged RCT, enquired how important they believed this trial to be and whether they would be happy to be randomised, or have their child randomised, to either group, following an explanation of the treatment procedures involved (e.g. intravenous access). As a check against the survey eligibility criteria, participants were asked whether they (or their child) had isolated *P aeruginosa *twice or more in the last 6 months.

Results from categorical data were presented as percentages in tables (including numerator and denominator), continuous data were presented as medians and ranges. No formal statistical testing was carried out on the data. To estimate the potential number of participants that would be eligible for a future trial, the median rate per annum of first or new growths was calculated from the clinician questionnaires and applied to the patient populations provided by the UK CF Trust, applying an estimated consent rate (based on consumer responses) to this figure would then give the potential number of eligible participants for the proposed trial.

## Results

For the clinician survey 63 clinicians from 57 CF centres throughout the UK were approached and 42 clinicians returned survey questionnaires, 33 online (from 31 independent centres) and an additional 9 received via postal survey following termination of the online access. This provided an overall response rate from clinicians of 70%. The majority of clinicians reported that they would treat a new isolation of *P aeruginosa *in a given individual in the same way that they treated their first ever isolation, routinely favouring oral ciprofloxacin in combination with nebulised colistin (Table 1). However, four clinicians reported that they would routinely treat first presentation of *P aeruginosa *with IV antibiotics in combination with colistin and 38 clinicians who completed the survey indicated that they would consider treatment of first or new growth of *P aeruginosa *with IV antibiotics under certain circumstances, particularly if the patient was clinically unwell.

**Table 1:** Reported treatment practice of clinicians in specialist CF centres

		Yes	No
Is the treatment of a new growth of *P aeruginosa *the same as the first ever isolation^a^		33/41 (80%)	8/41 (20%)

Routine treatment of patients who present with their first growth of *P aeruginosa*^b^	Oral ciprofloxacin for 3 weeks and nebulised colistin for 3 months	21/29 (72%)	8/29 (28%)
	
	Oral ciprofloxacin and nebulised colistin for 3 months	10/22 (45%)	12/22 (55%)
	
	IV antibiotics for 2 weeks and nebulised colistin for 3 months	4/18(22%)	14/18 (78%)
	
	Other	9/22 (41%)	13/22 (59%)

Is the first or a new *P aeruginosa *isolation *ever *treated with IV antibiotics and nebulised colistin rather than oral ciprofloxacin and nebulised colistin?		38/42 (90%)	4/42 (10%)

Likely to treat first or new growth of *P aeruginosa *with IV antibiotics when^b^	The patient is clinically unwell	35/35 (100%)	0/35 (0%)
	
	The patient has had a previous culture of *P aeruginosa*	13/24 (54%)	11/24 (46%)
	
	The patient has reduced lung function	28/31 (90%)	3/31 (10%)
	
	Other reason	14/21 (67%)^c^	7/21 (33%)

^a^1 respondent did not complete this question

^b^No responses were compulsory for this question and clinicians were able to select more than one option

^c ^5 other reasons related to clinical deterioration; 3 to ciprofloxacin resistance and 2 to patient preference/adherence

Clinicians' perceptions of the available evidence for oral antibiotic treatment, in combination with nebulised colistin, and their views on the efficacy of IV versus oral antibiotics as an eradication therapy were explored. When asked how good they thought the evidence was to support the treatment of first or new growth of *P aeruginosa *with oral ciprofloxacin and nebulised colistin, none thought it to be excellent, 46% thought it to be good, 42% fair, 10% poor and 2% replied 'don't know'. Over half (21 [53%]) of clinicians responded 'don't know' when asked to express an opinion about whether IV antibiotics are more effective than oral ciprofloxacin (each in combination with nebulised colistin for a period of three months) in eradicating first or new growth of *P *aeruginosa; 11 (27%) thought they were more effective and eight (20%) that they were not. Twenty nine (74%) of the 39 clinicians who answered the question were prepared to recruit patients to the proposed trial, with only two (5%) stating that they would not do so, due to their own perception that oral treatment is effective. Eight respondents (21%) were unsure that they would recruit patients into the proposed trial and three respondents did not answer this question. Clinicians were asked for general comments about the design of the trial and whether they would suggest alternative primary outcome(s). A variety of suggestions were made, but there was overall agreement that the primary outcome should reflect growth of *P aeruginosa *from lower respiratory tract cultures at some time point.

The median number of patients that were registered from the 31 independent centres that replied was 125 (range 35 to 380). The median percentage of patients who had never grown *P aeruginosa *was 26% (range 6% to 63%). The median rate per annum for the number of first or new growths of *P aeruginosa *in adults was 3% (range 1% to 9%) and in children was 10% (range 3% to 23%).

106 consumers from six UK CF centres completed and returned survey questionnaires. Centres did not report on the number of consumers who refused to accept a questionnaire when offered, therefore it is not possible to draw assumptions about potential bias which might have occurred as a result of refusals, however of those questionnaires issued only one was returned not completed to researchers. Sixty-eight (64%) of the consumer surveys were received from exclusively paediatric centres and 60 (56%) of consumer respondents were parents. Median age of the patient with CF was 10 years (Range 1 to 51 years); 62 (58%) were female. Forty (37%) patients had never grown *P aeruginosa *from a cough swab or sputum sample and two did not respond to the question. Of the 64 respondents who had reported previous infection with *P aeruginosa*, 22 (34% of respondents) had experience of being treated for a first or new growth with intravenous (IV) antibiotics plus colistin (Table 2).

**Table 2:** Treatment experiences of 64 participants who had previously isolated Pseudomonas from a cough swab or sputum sample.

		Yes	No	Don't know
Have Isolated P. *aeruginosa* ≥ 2 times in last 6 months^a^		5/63 (8%)	55/63 (87%)	3/63 (5%)

Treated for first or new growth with oral ciprofloxacin plus colistin		53/64 (83%)	6/64 (9%)	5/64 (8%)

Treated for first or new growth with IV antibiotic plus colistin		22/64 (34%)	37/64 (58%)	5/64 (8%)

		Treated for first or new growth with IV antibiotic plus colistin		
		
		**Yes**	**No**	**Don't know**

Treated for first or new growth with oral ciprofloxacin plus colistin	**Yes**	20	32	1
	
	**No**	1	4	1
	
	**Don't know**	1	1	3

^a^1 respondent did not complete this question.

When asked whether in their opinion oral or IV antibiotics would be better at eradicating P aeruginosa, about half of all consumers responded that they did not know (Table 3). Forty-five respondents (30 parents), out of 99, indicated that they would consider entry into the proposed RCT. The main reason given by consumers, who answered no to this question, was that they did not wish to participate in a trial. Other reasons included constraints on time, that they did not want unnecessary hospital admission and that they would not want to have IV treatment; two explicitly stated that they did not like needles. The main reason stated by consumers who would consider trial entry was to help in future research. Eighteen of the affirmative respondents had no previous experience of *P aeruginosa *infection.

**Table 3:** Consumer opinions about efficacy of P *aeruginosa *treatment

	Definitely better	Possibly better	No difference	Possibly worse	Definitely worse	Don't know
Is oral ciprofloxacin better than IV antibiotics at getting rid of pseudomonas?^a^	7 (7%)	17 (16%)	4 (4%)	19 (18%)	6 (6%)	51 (49%)

Are IV antibiotics better than oral ciprofloxacin at getting rid of pseudomonas?^b^	10 (10%)	29 (28%)	3 (3%)	5 (5%)	3 (3%)	53 (51%)

^a ^2 participants did not respond to this question

^b ^3 participants did not respond to this question

The total adult population reported by clinicians who responded to the survey was 1210 (9 centres) and the total paediatric population was 2676 (20 centres). Two responses were received from centres with mixed adult and paediatric populations for whom we could not derive the separate population sizes, therefore they are excluded from estimates. To estimate the eligible population for the proposed trial we applied the median rate per annum of first or new growths of *P aeruginosa *to each of these populations individually using the respective rates presented above (3% and 10% for adults and children respectively), which provided an estimate of 30 adults and 262 children per annum, from these centres, who would be eligible for recruitment. The UK CF Trust provided data for patients from 21 adult centres, 27 paediatric centres and two centres with both children and adults, who were able to provide figures for both groups. Applying the above rates to the population data provided by the UK CF Trust provides a conservative estimate of adults and children of 91 and 450 respectively per annum. If the consent rate was estimated to be 45% then the number of eligible patients per annum who would be willing to take part in a study, as presented in our survey would be approximately 41 adults and 203 children per annum.

## Discussion

The key finding from this feasibility study is that, although management of first or new presentations of *P aeruginosa *isolations in the UK is generally in line with UK CF Trust guidelines[6], there is considerable uncertainty amongst clinicians about whether the systemic therapy used in this regimen should be with IV or oral anti-pseudomonal antibiotics. Reflecting this doubt, all clinicians stated that they would consider using IV antibiotics in specific circumstances. When consumers were asked their views about the effectiveness of IV or oral treatment, they showed even greater uncertainty, with only a minority stating that one treatment was "definitely" better or worse.

The majority of clinicians, who completed this survey, indicated that they would be prepared to consider entering patients under their care into an RCT investigating these alternative treatments. There was also reasonable support for such a trial in the consumer population, with 45% reporting that they would consider entry. This study also provided important information about reasons why patients may not wish to enter this trial (e.g. potential inconvenience of hospital admission for IV treatment), some of which should be considered in trial design (e.g. provision of IV therapy at home, where appropriate).

The funding body was concerned about whether, with these estimates of clinician participation in recruitment and the willingness of potential participants to be enrolled in the trial, sufficient numbers could be randomised to address the objectives. Therefore we estimated the sample size of this study, based on each of the two primary outcomes which we had proposed. For the primary outcome "time to chronic *P aeruginosa *infection", data were examined from a Danish study[4] which reported that, over a three and a half year period, seven of 48 (15%) CF patients with lower airways cultures of *P aeruginosa*, who were treated with colistin inhalation and oral ciprofloxacin for between three weeks and three months developed chronic *P aeruginosa *colonisation compared with 19 of 43 (72%) historical controls. This suggested that in the patients who were treated with colistin inhalation and oral ciprofloxacin, chronic infection would be prevented or delayed in 85% of patients. To detect an increase from 85% to 90% or 95% free of chronic infection in the IV group would require 690 or 146 per group respectively for 80% power at the 5% significance level. To estimate the sample size based upon a primary outcome of 'eradication of infection', we estimated the initial eradication rate, using data from an observational study [7] where the protocol used colistin inhalation and oral ciprofloxacin achieved eradication (three consecutive negative respiratory *P aeruginosa *cultures and negative serum antibody titres within a six month period) in 47/58 (81%) patients. If the initial eradication rate in the comparator arm is assumed to be 80% to detect an increase to 90% with IV antibiotics, for 80% power at 5% significance level, would need 219 per group, or 263 per group allowing for a 20% dropout. These sample size calculations should be considered in the context of a trial which would run over at least two years and where the total number of potentially eligible patients in all of the UK per year was about 250.

Overall, clinical trials which have investigated routine therapies in patients with cystic fibrosis are relatively few and of inadequate design[8,9]. Although, a number of recent trials have addressed the benefits of existing therapies[10,11] and this picture is slowly improving[12], there are still considerable uncertainties about the effectiveness of many widely used, expensive and often invasive treatments for people with CF. CF patients and families are generally very well informed about their disease and its treatment, but this is the first study, of which we are aware, which has formally sought their views about acceptability of a proposed clinical trial. This study was conducted by staff at the MCRN Co-ordinating Centre and CTU; one of the central principles of MCRN is that children and families should be involved in all aspects of research which is relevant to their needs[13,14]. Although it may be argued that conducting a feasibility survey could delay the initiation of an important RCT, exploring opinions of clinicians and consumers in this way enables an informed decision to be made about the acceptability and feasibility of a proposed RCT. This feasibility study was conducted over a period of approximately six months and cost around £16,000; the time and financial requirements are minimal when compared to the potential expenditure associated with the initiation of a clinical trial that does not recruit enough patients to answer the clinical question.

Questionnaire surveys such as these have recognised limitations and every attempt was made to maintain anonymity of respondents in order to obtain true opinions. Although consumers completed questionnaires during routine clinic visits they were assured that their local team could not access responses as they were able to return their anonymously completed survey directly to the researchers. Additionally, the clinician surveys were completed remotely and anonymously. Despite this, there is a possibility that whilst clinicians may have indicated a general willingness to randomise their patients, they may be reluctant to do so in specific clinical circumstances. Similarly, patients/families previous experience of eradication therapy may influence their responses and their willingness to participate in a trial and these considerations are a reflection of the real life challenges facing clinicians conducting clinical trials.

This survey indicated that it was feasible to consider the initiation of a randomised controlled trial investigating eradication therapy to treat *P aeruginosa *in patients with CF. We have reported these findings to the funders of the feasibility study, The NIHR Health Technology Assessment Programme, who subsequently took the decisions to commission this trial and subsequently to award funding to a group of investigators to run this trial in the UK http://www.hta.ac.uk/1763.

## Conclusions

This study confirmed the uncertainty amongst clinicians and patients about the optimal way to treat first or new isolation of *P aeruginosa *from the airways of patients with CF and provided sound rationale for the clinical trial. It provided an estimate of the proportions of potentially eligible patients, of patients and clinicians who would be prepared to participate in such a RCT and aspects of trial design which might make it more acceptable. This information is essential to informing the design of the trial and whether it would be feasible, even when conducting across the United Kingdom. Such feasibility studies are particularly important for trials with patients with rare diseases.

## List of Abbreviations

CF: Cystic fibrosis; CTU: Clinical Trials Unit; MCRN: Medicines for Children Research Network; RCT: Randomised controlled trial.

## Competing interests

MCRN CTU (Paula Williamson, Ashley Jones and Helen Hickey) are collaborating with clinicians conducting the TORPEDO-CF trial of optimal therapy for pseudomonas eradication in cystic fibrosis. Ashley Jones is an investigator in the TORPEDO-CF trial.

## Authors' contributions

HH (Senior Trials Manager) contributed to the design of the questionnaires, coordinated the study and was involved in the writing of the report, AJ (Senior Statistician) analysed the survey results and was involved in the writing of the report, RS (Professor of Paediatric Medicine) contributed to the design of the study and survey questionnaires and was involved in the writing of the report, PW (Professor of Medical Statistics) contributed to the design of the study and survey questionnaires, supervised the data analysis and was involved in the writing of the report. WL (Professor of Respiratory Paediatrics), put forward the concept of comparing oral and IV therapy in CF to the Health Technology Assessment Programme and contributed to the design of this study, the design of the questionnaires and the editing of the report.

## Supplementary Material

Additional file 1 Clinician Questionnaire. Click here for file

Additional file 2 Consumer Questionnaire. Click here for file
