# Supplementary material for: Feasibility study to inform the design of a randomised controlled trial to eradicate Pseudomonas aeruginosa infection in individuals with Cystic Fibrosis
Source: Trials. 2010 Feb 5;11:11. doi: 10.1186/1745-6215-11-11 (PMC2832639; doi:10.1186/1745-6215-11-11)
Supplement: Additional file 1 — Clinician Questionnaire. [file 1745-6215-11-11-S1.DOC]

**Clinician Questionnaire**

# Prevention of colonisation with Pseudomonas *aeruginosa* for

# Cystic Fibrosis patients

# NHS R&D Health Technology Assessment Programme 06/21/01 Brief

The HTA programme requests a report on the feasibility of a trial to answer the question: “What is the clinical and cost effectiveness of intravenous antibiotics after the first *Pseudomonas aeruginosa* culture in delaying the re-infection of the lungs of cystic fibrosis sufferers?”

The Medicines for Children Clinical Trials Unit (MCRN CTU) is therefore conducting this survey, on behalf of the Medicines for Children Research Network and the Cystic Fibrosis Trust, to explore current clinical practice in this area and opinions of clinicians about such a trial. A survey of representative samples of adults and young people (aged 13 years plus) who have cystic fibrosis, and parents of children aged less than 13 years, will also be undertaken.

# Trial to be considered

The HTA envisage the proposed trial will take the form of a randomised controlled trial (RCT). Patients with cystic fibrosis, who are not colonised with *Pseudomonas aeruginosa* as defined by CF Trust guidelines (*Pseudomonas aeruginosa* infection in people with cystic fibrosis 2004), from whom *P.aeruginosa* is isolated, will be randomised to receive either 2 weeks of anti-pseudomonal intravenous antibiotics or current standard treatment with oral ciprofloxacin. Both groups would receive nebulised colistin for a maximum of 3 months. Discussions with colleagues involved in the treatment of cystic fibrosis patients indicate that the design of the RCT should have the following features:

- Patients and clinic teams will be unblinded but assessment of outcomes would be by investigators blinded to the treatment received
- Experimental arm comprising 2 weeks treatment with IV antibiotic (this being an agent effective against *P. aeruginosa*, whatever is current practice in the given clinic)
- Standard arm comprising initial 3 weeks treatment, continued to a maximum of 3 months treatment with oral ciprofloxacin.
- BOTH arms to include 3 months treatment with nebulised colistin
- Follow-up would be 2 years from randomisation, scheduled to coincide with routine clinic attendance.
- The aim would be to recruit each patient only once (i.e. not to assess the input of repeated regimens of treatment on individual patients)
- Lower respiratory tract cultures (sputum, oropharyngeal or bronchoalveolar lavage [BAL]) would be taken from patients at least at month’s one, two and three following initiation of treatment and two monthly thereafter.

**Suggested primary outcome measures:**

1. Time to next growth of *P. aeruginosa* following eradication therapy.
2. Eradication of *P aeruginosa* from the lower respiratory tract, defined as: No isolation of *P aeruginosa* from any lower respiratory tract culture (sputum, oropharyngeal or BAL) over a 2 year period (with screening taking place as detailed above).

| **1.** | What is your current clinical practice for the treatment of patients with cystic fibrosis who present with their **first** growth of Pseudomonas aeruginosa from a cough swab or sputum sample? | | | |
| --- | --- | --- | --- | --- |
|  | **1a.** Routinely treat with oral ciprofloxacin for 3 weeks and nebulised colistin for 3 months. If repeat culture is negative at 3 weeks, no further ciprofloxacin is given | | Yes |  |
|  |  |  | No |  |
|  |  | |  |  |
|  | **1b.** Routinely treat with nebulised colistin and oral ciprofloxacin, both therapy’s for 3 months | | Yes |  |
|  |  |  | No |  |
|  |  | |  |  |
|  | **1c.** Routinely treat with IV antibiotics for 2 weeks and nebulised colistin for 3 months | | Yes |  |
|  |  |  | No |  |
|  |  | |  |  |
|  | **1d.** Other? | | Yes |  |
|  |  |  | No |  |
|  | If you answered ‘Yes’ to 1d, please specify: | |  |  |
|  |  | | | |
|  |  | | | |
|  |  | | | |
| **2.** | When a patient with cystic fibrosis presents with a **new** growth of Pseudomonas aeruginosa from a cough swab or sputum sample, i.e. after previous successful eradication therapy), do you treat them in the same way that you treat a patient who presents with their **first growth** of P aeruginosa? | | Yes | Go to Q4 |
|  |  |  | No | Go to Q3 |
|  |  | |  |  |
| **3.** | What is your current clinical practice for the treatment of patients with cystic fibrosis who present with a **new** growth of P aeruginosa from a cough swab or sputum sample (i.e. after previous successful eradication therapy)? | | | |
|  | **3a.** Routinely treat with oral ciprofloxacin for 3 weeks and nebulised colistin for 3 months. If repeat culture is negative at 3 weeks, no further ciprofloxacin is given | | Yes |  |
|  |  |  | No |  |
|  |  | |  |  |
|  | **3b.** Routinely treat with nebulised colistin and oral ciprofloxacin, both therapy’s for 3 months | | Yes |  |
|  |  |  | No |  |
|  |  | |  |  |
|  | **3c.** Routinely treat with IV antibiotics for 2 weeks and nebulised colistin for 3 months | | Yes |  |
|  |  |  | No |  |
|  |  | |  |  |
|  | **3d.** Other? | | Yes |  |
|  |  |  | No |  |
|  | If you answered ‘Yes’ to 3d, please specify: | |  |  |
|  |  | | | |
|  |  | | | |
|  |  | | | |
|  |  | | | |
| **4.** | Do you **ever** treat first or new growth of Pseudomonas aeruginosa with IV antibiotics in combination with nebulised colistin, rather than oral ciprofloxacin in combination with nebulised colistin? | | Yes | Go to Q5 |
|  |  |  | No | Go to Q6 |
|  |  | |  |  |
| **5.** | Are you likely to treat first or new growth of Pseudomonas aeruginosa with IV antibiotics when: | | | |
|  | **5a.** The patient is clinically unwell? | | Yes |  |
|  |  |  | No |  |
|  |  | |  |  |
|  | **5b.** The patient has had a previous positive culture of Pseudomonas aeruginosa? | | Yes |  |
|  |  |  | No |  |
|  |  | |  |  |
|  | **5c.** The patient has reduced lung function, e.g. FEV_1_ <70% | | Yes |  |
|  |  |  | No |  |
|  |  | |  |  |
|  | **5d.** Other reason? | | Yes |  |
|  |  |  | No |  |
|  | If answered ‘Yes’ to 5d, please specify: | |  |  |
|  |  | | | |
|  |  | | | |
|  |  | | | |
| **6.** | How good do you think the evidence is to support the treatment of first or new growth of Pseudomonas aeruginosa with oral ciprofloxacin and nebulised colistin? | Poor | | |
|  |  | Fair | | |
|  |  | Good | | |
|  |  | Excellent | | |
|  |  | Don’t know | | |
|  |  |  | | |
| **7.** | **7a** Do you think that IV antibiotics for 2 weeks, in combination with nebulised colistin for 3 months, may be more effective than oral ciprofloxacin in combination with 3 months of nebulised colistin for eradication of first or new growth of Pseudomonas aeruginosa? | Yes | |  |
|  |  | No | |  |
|  |  | Don’t know | |  |
|  |  |  | |  |
|  | **7b** How long do you think treatment with oral ciprofloxacin should routinely be continued for? | | | |
|  |  | | | |
|  |  |  | |  |
| **8.** | Please explain below the reason/s for your answers to question 7: | | | |
|  |  | | | |
|  |  | | | |
|  |  |  | |  |
| **9.** | Would you be prepared to recruit patients into a trial to compare oral and IV antibiotics, with both groups being given nebulised colistin for 3 months? | Yes | |  |
|  |  | No | |  |
|  |  | Don’t know | |  |
|  |  |  | |  |
| **10.** | Please explain below the reason/s for your answer to question 9 | | | |
|  |  | | | |
|  |  | | | |
|  |  | | | |
|  |  | | | |
| **11.** | **11a.** Would you suggest an alternative primary outcome to that suggested in the attached information? | | | |
|  |  | | | |
|  |  | | | |
|  |  | | | |
|  | **11b.** Do you have any comments about the trial design proposed in the attached information? | | | |
|  |  | | | |
|  |  | | | |
|  |  | | | |
|  |  | |  |  |
|  | If the data are available to you, we would be very grateful if you could also provide the following information: | | | |
|  |  | | | |
| **12.** | In total, how many patients with cystic fibrosis are registered at your centre? | |  |  |
|  |  | |  |  |
|  | Of this total: | |  |  |
|  | **12a.** How many are **currently** **not** colonised with *P aeruginosa*? | |  |  |
|  |  | |  |  |
|  | **12b.** How many **have never** grown *P aeruginosa* from lower respiratory tract culture? | |  |  |
|  |  | |  |  |
|  | **12c.** How many **first or new** growths of *P aeruginosa* occur in your clinic per annum? | |  |  |
|  |  | |  |  |
|  |  | |  |  |

**Thank you for taking the time to complete this survey. Please place in the enclosed stamped self addressed envelope for return to the MCRN CTU**
