# Supplementary material for: Feasibility study to inform the design of a randomised controlled trial to eradicate Pseudomonas aeruginosa infection in individuals with Cystic Fibrosis
Source: Trials. 2010 Feb 5;11:11. doi: 10.1186/1745-6215-11-11 (PMC2832639; doi:10.1186/1745-6215-11-11)
Supplement: Additional file 2 — Consumer Questionnaire. [file 1745-6215-11-11-S2.DOC]

**Consumer Questionnaire**

***Consumer Questionnaire***

**The Medicines for Children Research Network and Cystic Fibrosis Trust are carrying out a survey in your hospital clinic**

This is a survey conducted by the Medicines for Children Clinical Trials Unit on behalf of the Medicines for Children Research Network and the Cystic Fibrosis Trust. The survey will include adults and young people aged 13 years of age or more who have cystic fibrosis and also parents of children with cystic fibrosis who are under 13 years of age.

**Why are we carrying out this survey?**

People with cystic fibrosis are at risk of developing infection in their lungs. If this should happen then a common cause of the infection can be a bacterium, or germ, called Pseudomonas aeruginosa. When this is first found in the sputum or cough swab the usual treatment is that patients take an antibiotic (ciprofloxacin) by mouth for 3 weeks along with an inhaled (nebulised) antibiotic (colomycin). If this does not get rid of (eradicate) the Pseudomonas aeruginosa.the treatment may be continued for 3 months. This is an intensive treatment plan which aims to eradicate the Pseudomonas aeruginosa bacteria and prevent damage to the lung tissue. Following such treatment, Pseudomonas aeruginosa is considered to be successfully eradicated if cough swabs or sputum samples are negative for Pseudomonas aeruginosa at least 3 times in a row over a 6 month period. Re-infection with Pseudomonas aeruginosa after successful eradication would be regarded as a new infection and treated intensively as described for first infection.

It is widely accepted that intensive treatment is essential to try and get rid of the Pseudomonas aeruginosa bacteria from the lung and the treatment method described above has been used for several years. Even so, we do not know for sure if this is the best way possible to get rid of this type of infection.

The alternative treatment plan that is being considered would be an antibiotic given intravenously (IV), that is via a needle inserted into a blood vessel (vein) in the arm, or via a portacath if you have one, for 2 weeks along with nebulised colomycin for 3 months. Giving antibiotics intravenously may work better than the oral treatment, because it enables a larger dose of antibiotics to be delivered to the lungs, but we do not know if this treatment will work better in practice.

The only way to discover which is the best treatment plan is to compare them against each other in a research project called a clinical trial. Patients who enter a clinical trial of this type would receive either one or other of the treatments and would be checked closely as the treatment is given and in the months afterwards to see which plan is better at getting rid of the infection in the first place and also in helping to avoid re-infection. The difference between the two treatment groups would be that one group would receive ciprofloxacin, taken twice a day by mouth, for a maximum of 3 months. The other group would receive an antibiotic via a vein (or portacath), generally three times a day, for 2 weeks. Both groups would receive nebulised colomycin for 3 months. Ciprofloxacin is taken by mouth and therefore can be given at home but if patients are to be given intravenous antibiotics and have never been treated with them before then this course of treatment is given in hospital. Like all medicines, these antibiotics can cause side effects in some people and some people may find they have an allergy to them. The most common side effects associated with antibiotic treatments are rashes and tummy upsets such as feeling sick (nausea), being sick (vomiting) or loose stools (diarrhoea).

Before this clinical trial can be planned in any detail we need to know what you think about it, therefore we have created the attached survey which we would like you to complete. Your answers to the questions in this survey are very important to us.

**Do I have to take part in this survey?**

You do not have to take part in this survey. If you choose not to take part you do not have to give a reason. Simply hand it back to the clinic staff without completing it.

**If I want to take part in this survey, what will I have to do?**

You are asked to answer the 10 questions contained in the survey.

**Will anyone know I'm doing this?**

Your answers will be treated in total confidence. The clinic staff will know you have been asked to take part in this survey, but when you have completed the questionnaire and placed it in the sealed envelope provided it is returned directly to us in the Medicines for Children Clinical Trials Unit. The questionnaire asks for some details about yourself, but we will not be able to identify you from any of the information that you provide.

| **1.** | Are you (please tick): | | | | |  | |  | | | |
| --- | --- | --- | --- | --- | --- | --- | --- | --- | --- | --- | --- |
|  | an individual with cystic fibrosis (adult or young person aged 13 years or more)? | | | | | | |  | | |  |
|  | or the parent of a child with cystic fibrosis who is aged less than 13 years? | | | | | | |  | | |  |
|  |  | | | | |  | |  | | | |
| **2.** | Please provide the following details about yourself (or your child if you are a parent) | | | | | | |  | | | |
|  | Age at last birthday: |  | (in years) | Gender: | Male | Female | | |  | | |
|  |  | | | | |  | |  | | | |
| **3.** | Have you/your child ever had Pseudomonas aeruginosa grown from a cough swab or sputum sample? | | | | | Yes | |  | | Go to question 4 | |
|  |  |  |  |  |  | No | |  | | Go to question 7 | |
|  |  |  |  |  |  | Don’t know | |  | | Go to question 7 | |
|  |  | | | | |  | |  | |  | |
| **4.** | Have you/your child grown Pseudomonas aeruginosa from a cough swab or sputum sample two or more times in the past six months? | | | | | Yes | |  | |  | |
|  |  |  |  |  |  | No | |  | |  | |
|  |  |  |  |  |  | Don’t know | |  | |  | |
|  |  | | | | |  | |  | |  | |
| **5.** | Have you/your child ever been treated for first or new growth of Pseudomonas aeruginosa using nebulised colomycin and oral ciprofloxacin? | | | | | Yes | |  | |  | |
|  |  |  |  |  |  | No | |  | |  | |
|  |  |  |  |  |  | Don’t know | |  | |  | |
|  |  | | | | |  | |  | |  | |
| **6.** | Have you/your child ever been treated for first or new growth of Pseudomonas aeruginosa using nebulised colomycin and IV antibiotic? | | | | | Yes | |  | |  | |
|  |  |  |  |  |  | No | |  | |  | |
|  |  |  |  |  |  | Don’t know | |  | |  | |
|  |  | | | | |  | |  | |  | |
| **7.** | The clinical trial being thought about would compare IV antibiotics with oral antibiotics for treating first or new growth of Pseudomonas aeruginosa. Both groups will receive nebulised colomycin. We would like to know your opinion of oral versus IV antibiotic treatment. | | | | | | | | | | |
|  |  | | | | | | | | | | |
|  | **7a.** Do you think that oral ciprofloxacin is better than IV antibiotics at getting rid of the pseudomonas? | | | | | Definitely better | | | | | |
|  |  |  |  |  |  | Possibly better | | | | | |
|  |  |  |  |  |  | No difference | | | | | |
|  |  |  |  |  |  | Possibly worse | | | | | |
|  |  |  |  |  |  | Definitely worse | | | | | |
|  |  |  |  |  |  | Don’t know | | | | | |
|  |  |  |  |  |  |  |  |  |  |  |  |
|  | **7b.** Do you think that IV antibiotics are better than oral ciprofloxacin at getting rid of the pseudomonas? | | | | | Definitely better | | | | | |
|  |  |  |  |  |  | Possibly better | | | | | |
|  |  |  |  |  |  | No difference | | | | | |
|  |  |  |  |  |  | Possibly worse | | | | | |
|  |  |  |  |  |  | Definitely worse | | | | | |
|  |  |  |  |  |  | Don’t know | | | | | |
| **8.** | Would you like to add any extra comment about your answers to questions 7a or 7b? | | | | | | | | | | |
|  |  | | | | | | | | | | |
|  |  | | | | | | | | | | |
|  |  | | | | | | | | | | |
| **9.** | Would you consider entry, for yourself/your child, into a clinical trial that is investigating treatment of Pseudomonas aeruginosa, such as the one described in the attached information? | | | | | | Yes |  | | | |
|  |  |  |  |  |  |  | No |  |  |  |  |
|  |  | | | | | |  |  | | | |
| **10.** | Please explain below the reason/s for your answer to question 9: | | | | | | | | | | |
|  |  | | | | | | | | | | |
|  |  | | | | | | | | | | |
|  |  | | | | | | | | | | |
|  |  | | | | | |  |  | | | |
|  | **Thank you for taking the time to complete this survey. Please place the questionnaire in the envelope provided and hand it in to clinic staff.** | | | | | | | | | | |
